# Bidirectional Jump Point Search Path-Planning Algorithm Based on Electricity-Guided Navigation Behavior of Electric Eels and Map Preprocessing

**DOI:** 10.3390/biomimetics8050387

**Published:** 2023-08-25

**Authors:** Hao Gong, Xiangquan Tan, Qingwen Wu, Jiaxin Li, Yongzhi Chu, Aimin Jiang, Hasiaoqier Han, Kai Zhang

**Affiliations:** 1 Changchun Institute of Optics, Fine Mechanics and Physics, Chinese Academy of Sciences, Changchun 130033, China; 2 Research Center for Materials and Optoelectronics, University of Chinese Academy of Sciences, Beijing 100049, China; 3 CAS Key Laboratory of On-Orbit Manufacturing and Integration for Space Optics System, Chinese Academy of Sciences, Changchun 130033, China

**Keywords:** electricity guided, grid map, inflection points, map preprocessing, path planning, rewiring

## Abstract

The electric eel has an organ made up of hundreds of electrocytes, which is called the electric organ. This organ is used to sense and detect weak electric field signals. By sensing electric field signals, the electric eel can identify changes in their surroundings, detect potential prey or other electric eels, and use it for navigation and orientation. Path-finding algorithms are currently facing optimality challenges such as the shortest path, shortest time, and minimum memory overhead. In order to improve the search performance of a traditional A* algorithm, this paper proposes a bidirectional jump point search algorithm (BJPS+) based on the electricity-guided navigation behavior of electric eels and map preprocessing. Firstly, a heuristic strategy based on the electrically induced navigation behavior of electric eels is proposed to speed up the node search. Secondly, an improved jump point search strategy is proposed to reduce the complexity of jump point screening. Then, a new map preprocessing strategy is proposed to construct the relationship between map nodes. Finally, path planning is performed based on the processed map information. In addition, a rewiring strategy is proposed to reduce the number of path inflection points and path length. The simulation results show that the proposed BJPS+ algorithm can generate optimal paths quickly and with less search time when the map is known.

## 1. Introduction

Path planning is the search for a safe path without collision from start to target. It needs to satisfy some optimal metrics (e.g., search time, path length, memory overhead, etc.) [1], and there may be trade-offs between these metrics. Path planning is not only a theoretical study, but it also plays an important role in many fields. This task is requisite to artificial intelligence applications, such as service robots, medical robots, farm robots, rescue robots, autonomous driving, unmanned ships, video games, etc. [2,3,4,5]. The success of task completion by the robot is directly influenced by the outcome of its path planning. If the path planning is not precise enough or if the path is not selected properly, it may result in the robot malfunctioning or even causing an accident. The necessity of path planning for robots is undeniable. However, the complexity of this problem requires a comprehensive consideration of various metrics and conditions to derive an optimal path that not only meets practical needs but also ensures safety.

Service robots, as the most common type of indoor mobile robots, have achieved great commercial success in recent years. However, the development of technology requires robots to pursue a faster speed to find the path. The requirements that a service robot should follow are shown below:

(1) The robot can run safely without collision from start to target.
(2) The robot is capable of real-time path planning.
(3) The robot should take the shortest and simplest path.
(4) The path-finding algorithm of the robot uses as little memory as possible.

However, it is difficult to satisfy all conditions in complex environments. The basic path planning consists of the following steps. First, the robot scans the environment boundaries and obstacles using sensors such as lidar and an odometer to construct a map of the environment [6]. Next, the robot uses path-finding algorithms for path planning. Finally, the robot works according to the planned path.

In order to perform path planning, it is necessary to create a map of the environment. The most popular and widely used method for representing the path-finding environment is the undirected uniform cost grid map, which distinguishes between movable space and obstacles and represents them numerically [6]. This brings convenience to path planning and has become an important research method adopted by many scholars.

The common mobile robot path-planning algorithms based on grid maps include Dijkstra [7], A* [8], Rapidly-exploring Random Trees algorithm [9], D-star [10], the Ant Colony Optimization Algorithm [11], Genetic Algorithm [12], Particle Swarm Optimization Algorithm [13], and so on. These algorithms have the characteristics of simple implementations and wide applicability, and they are common solutions for robot path planning. Different algorithms can be selected and optimized according to different task requirements and environmental characteristics.

The A* is a classic path-planning algorithm that uses a heuristic search to find the shortest path from the starting point to the target point in a quick and efficient manner without requiring a complete traversal of the entire map. It applies an evaluation function to rank the search frontier’s states, which takes into consideration the actual cost involved in moving from the starting point to the current state as well as the estimated cost of reaching the end point from the current state [8]. This heuristic technique allows the A* to find the path faster than Dijkstra and makes the A* optimized in terms of computational time consumption and space complexity.

A* is an effective path-planning algorithm. However, it has a drawback in that it requires more memory space and takes a longer time to maintain and store open and closed lists, especially in large-scale maps. This can cause the algorithm to slow down even in situations where real-time navigation is required [14]. For the drawbacks of the A*, the directions of its improvement are divided into heuristic functions and successor node generation.

The weighted heuristic search is a technique to improve the efficiency of the path search, and it is also widely used in heuristic search algorithms [1]. One of the most common is the weighted A*, which takes the heuristic function *f* = *g* + *wh*. It can balance the search speed and the accuracy of the optimal path by adjusting the heuristic function weight *w* [15]. In some scenarios where the solution time is tight, the map is large or the robot does not need to take the optimal path, the heuristic function weight can be used to obtain a faster search speed. The implementation of this strategy is relatively simple and only requires the inclusion of the weights of the heuristic function in the calculation of *f*. Pohl et al. proposed the weighted A* and experimentally obtained the best weight value 1 ≥ *w* ≥ 0.5 under the guaranteed shortest path [16]. Bulitko et al. proposed a learning real-time A* with a weight value of 2 ≥ *w* ≥ 0 to explore paths autonomously, but the drawback is that it over-explores and the paths are not necessarily optimal [17]. Wang et al. used exponential decay to weight the heuristic function of the A* to improve the computational efficiency of the algorithm [18].

**Figure 1:**
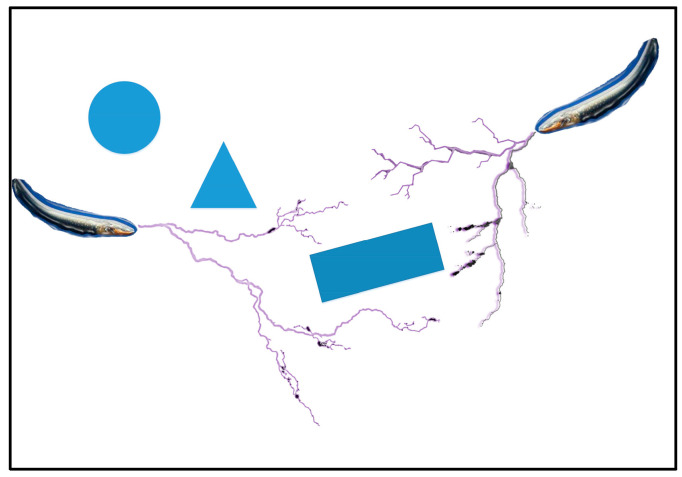
Electric eels guided by electric fields to find other companions.

The electricity-guided navigation behavior of electric eels brings ideas to the improvement of heuristic functions. Electric eels are able to find others in murky waters by using their electrical organs. Figure 1 shows electric eels guided by electric fields to find other companions. The electric organs of the electric eels can detect low-frequency electric signals in the surrounding environment [19]. When two electric eels want to find each other, they navigate by emitting electric field signals and sensing changes in the electric field around them. One emits a low-frequency electric signal, which the other is able to perceive in order to determine the direction and track the source of the signal. By constantly interacting with each other in emitting and sensing the electric field signals, the two electric eels can locate and find each other [20]. They use the high sensitivity and precision of their electrosensory organs and their ability to perceive changes in the electric field to navigate and communicate in the water. This ability to navigate by electric induction plays an important role in activities such as reproduction and communication [21]. It also provides inspiration for bionic research to develop novel sensory technologies and navigation systems to improve the ability of robots to locate and navigate in specific environments. Specifically, the behavior of sensing low-frequency electric signals is useful for improving the heuristic function.

For successor node generation, it has been discovered by some scholars that successor nodes do not always select neighboring nodes in the eight cardinal directions during path planning. In light of this, Harabor et al. introduced the JPS which selectively adds jump points to the open list [22]. This approach helps to eliminate extraneous nodes, expedite the path search, and minimize memory costs. Since the process of generating jump points is time consuming, Harabor et al. proposed the JPS+, which speeds up the path search by preprocessing the map [23]. Nonetheless, the search for jump points remains complicated. Nobes et al. have applied the JPS to 3D environments [24]. Meanwhile, Su et al. have developed an algorithm for artificial field-guided jump point research [25], which can decrease the number of node searches but may incur local extrema. On the other hand, Huang et al. have leveraged dynamic weighted optimization of the heuristic function to enhance the speed performance of the JPS [26].

The JPS provides a novel technique for solving the single-body path-finding problem with proven convergence and path minimization [22]. The aim of this study is to reduce the search time, the search space and the complexity of the algorithm implementation. To achieve these goals, this paper presents a bidirectional JPS based on map preprocessing and an improved jump point screening strategy. The proposed algorithm is able to efficiently reduce the path search time and algorithm implementation complexity within a known static environment. The main innovations of this algorithm are as follows:

(1) An improved heuristic function is proposed, which can speed up the path search efficiency and reduce the number of path search points while maintaining the shortest path.
(2) To reduce algorithm implementation complexity in jump point screening, a new jump point screening strategy is proposed. This involves converting jump points into turning points at obstacles, thereby decreasing the number of unnecessary jump points on the map and accelerating the jump point screening process.
(3) In order to decrease the number of visited nodes, the neighborhood search principle is modified to a five-domain search. This more compact approach to searching improves the efficiency of the target point search.
(4) To further optimize the search efficiency, a bidirectional search strategy is introduced. In this approach, JPS+ search is performed from both the starting point and the goal point, and it is guided by the sequence of preprocessed jump points. This method increases the search efficiency of the algorithm.
(5) In order to reduce the number of inflection points and the path length in the path nodes, a rewiring strategy is presented in this study. The initial path may contain a large number of inflection points. Therefore, the parallelogram rewiring strategy is utilized to prune the path nodes.

This paper consists of five sections. Section 1 serves as an introduction, providing a brief overview of the research background and the importance of path-planning algorithms. Section 2 discusses previous research results and problems in path-planning algorithms. Section 3 presents the proposed improved algorithm and elaborates on the specific strategies introduced in this paper, such as jump point screening, heuristic function, bidirectional search and rewiring. Section 4 focuses on experimental simulations, where path-planning experiments are performed using different environmental maps. The results are discussed and analyzed in detail. The conclusions are summarized in Section 5.

## 2. Related Work

This section presents the standard model of the JPS: a path-planning algorithm for grid maps based on the A* proposed by Harabo et al. in 2011. Compared to the traditional A*, the JPS speeds up the search by skipping useless points [14].

### 2.1. Grid Map Building

The method of building grid maps based on sensor data was originally proposed by Moravec and Elfes [27], which is widely used in the field of robotics. The method constructs a grid map through the following steps, including acquiring sensor data, constructing the original grid map, correcting the map, optimizing the map, and storing the map [28]. The method can effectively construct a grid map based on sensor data to support robot navigation and autonomous control.

In commonly used grid maps, each grid cell is assigned either a free space state (represented by 0) or an obstacle state (represented by 1). By partitioning the environment into individual grid cells, the path-planning algorithm can effectively represent the complex physical environment and provide critical information, such as the relative position and distance between individual grid cells. This grid cell partitioning method is both simple and intuitive, enabling it to effortlessly reflect diverse characteristics of different regions in the map and the relationships between these regions. Due to its versatility, the approach is widely used in the path planning of various mobile robots. In addition, the grid map implementation can be adjusted to suit varying scales of physical environments by adapting the grid cell size, allowing for flexible and efficient path-planning applications. Therefore, the path-planning method based on grid maps holds significant value and importance in addressing practical problems.

### 2.2. Original Algorithm Model

The center idea of the JPS is to use jump points to decrease the search space so as to achieve efficient path planning. The jump point is used to skip some intermediate nodes directly in the search process and directly search the key node where the target node is located. During the search, if the current node is not a jump point, it can be directly skipped, and only the jump point is searched. Alternatively, nodes that are not the jump points can be searched if necessary. This method can greatly reduce the search complexity and improve the search efficiency, especially for large and complex maps with good applicability.

The path finding using JPS starts with the starting point as the root node. Then, the search is extended by the jump points using heuristic search. If an obstacle or boundary is encountered during the search, the next jump point needs to be found to continue the search. Finally, when the search reaches the target point, a shortest path from the starting point to the target point can be obtained by backtracking.

**Figure 2:**
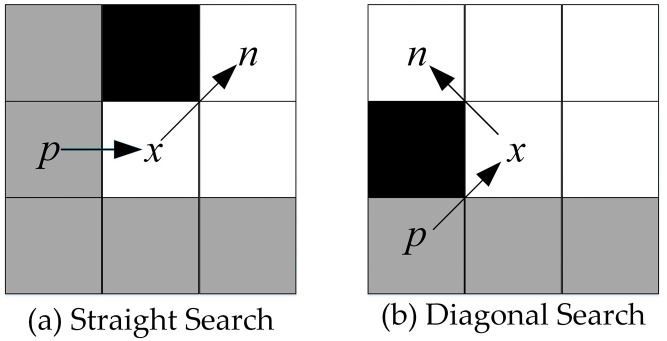
Forced neighbor.

The JPS algorithm utilizes the heuristic function *f* = *g* + *h* inherited from A*, where *f* represents the total cost, *g* represents the actual cost, and *h* represents the estimated cost. Distance calculations are typically performed using Euclidean, Manhattan, or Chebyshev distances. However, in contrast to A*, JPS extends nodes in the form of jump points with specific rules governing the screening process for these points:

Definition of Forced Neighbor: Node *n* is said to be a forced neighbor of *x* if there are obstacles among its eight neighbors and the distance cost of *x*’s parent node *p* arriving at *n* via *x* is smaller than the distance cost of any path to *n* that does not arrive via *x*. Figure 2 shows the schematic diagram of forced neighbors for straight search and diagonal search.

Definition of Jump Point: The jump point must be in the search direction. There are three types of jump points for the JPS:

(1) Nodes with forced neighbors;
(2) Target points;
(3) Points in the diagonal direction of node *x* that satisfy conditions 1 and 2 for a diagonal search.

In the third point, assuming an oblique search to the upper right, the straight search conducted by node *x* is limited to the right and up (vector decomposition of the diagonal search vector), and no search is conducted in the left and down directions, which is called expansion in the inherent straight direction during the diagonal search.

**Figure 3:**
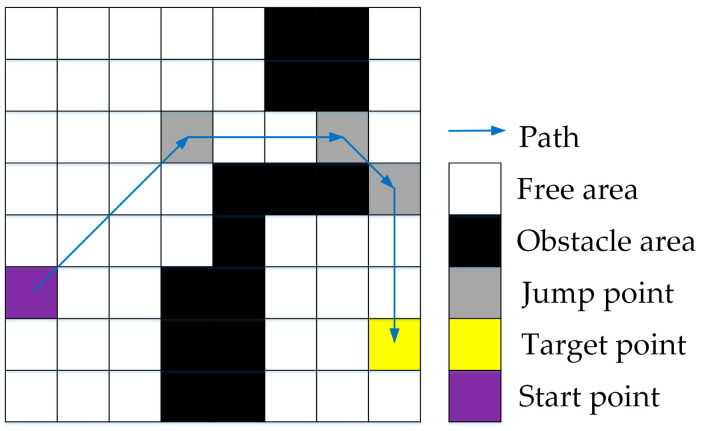
Path planning based on JPS.

Figure 3 shows an example of path planning based on the JPS. Although the JPS speeds up the search by filtering jump points, the process of generating jump points takes up a lot of memory space and computing resources, which still costs a lot of time and space.

## 3. Improved Algorithm

The results of the paper [14] show that the JPS spends about 90% of its time on the generation of successor nodes. Therefore, improving the efficiency of the JPS depends on how rapidly the successor nodes can be generated. A bidirectional JPS+ algorithm for map preprocessing is proposed, which is based on an improved jump point screening strategy that accelerates the path search.

### 3.1. Improved Heuristic Function

A* and JPS both use traditional heuristic function.

(1) f(s)=g(s)+h(s)

where *g*(*s*) is the cost value incurred to reach *s*, *g*(*s*) = *g*(*s* − 1) + *d*(*s* − 1,*s*), *h*(*s*) is the heuristic cost of reaching the target node *T* from *s*, and *h*(*s*) = *d*(*s*,*T*), *d* using Euclidean distance. The heuristic function *h* satisfies *h*(*s*) ≤ *d*(*s*,*s*′) + *h*(*s*′), and *s*′ is a node in the subsequent path.

**Figure 4:**
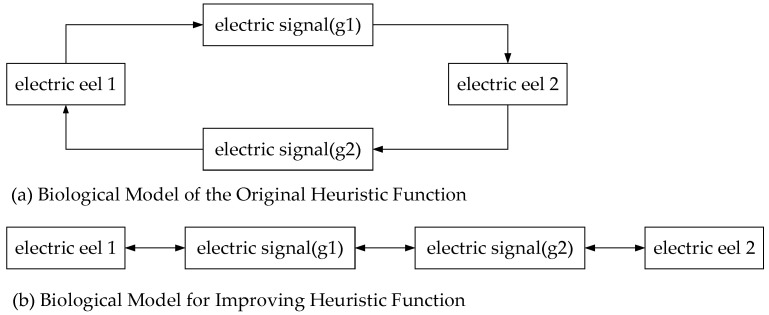
Biological principles.

The biological model for the heuristic function is shown in Figure 4. The biological model of the original algorithm can be described as two electric eels looking for each other’s initial position, as shown in Figure 4a. However, this ignores the heuristic function of the other’s current position and does not offer the greatest advantage of bidirectional search. By constantly emitting and perceiving weak electric field signals, the two eels can locate each other and find each other quickly. Referencing the electric eel model, in order to expand the advantages of bidirectional search, the design of the heuristic function should consider the current position of the other in the path search process. Formula (2) shows the improved heuristic function.

(2) $$f=g_{1}+g_{2}+h\prime$$

where *g*_1_ is the forward actual cost of the current point *s*, *g*_2_ is the real cost corresponding to the point with the minimum value of *f* when searching in reverse, and *h*′ is the distance between point *s* and point *s*′.

### 3.2. Improved Jump Points Screening Strategy

**Figure 5:**
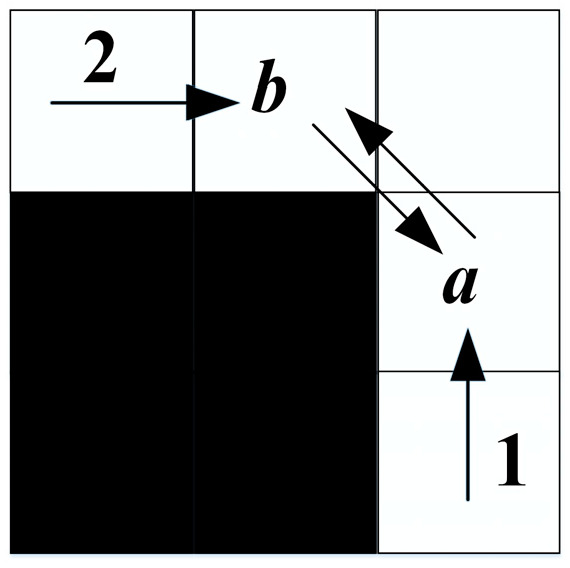
Jump point strategy.

In most cases, the JPS algorithm operates in pairs of jump points. For instance, as shown in Figure 5, when searching in direction 1, node *a* is identified as a jump point whereas node *b* is considered a forced neighbor. Conversely, when searching in direction 2, node *b* is a jump point while node *a* is regarded as a forced neighbor. While increasing the number of jump point types can potentially shorten the path length, it also results in a more complex algorithm implementation, greater path danger, and increased search time. Jump points are replaced with inflection points in this study, resulting in a reduced number of jump points and a faster path search.

**Figure 6:**
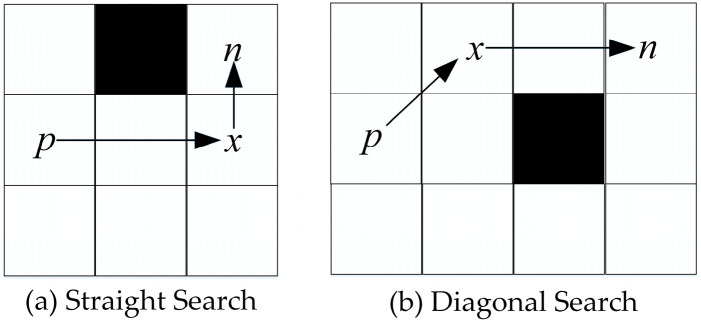
Improved jump point strategy.

(1) In straight search, the JPS replaces jump points with inflection points. Inflection points refer to locations where two paths intersect horizontally and vertically, which is accompanied by obstacles. As shown in Figure 6a, node *x* represents an inflection point.
(2) In diagonal search, if there is an inflection point in the intrinsic straight line direction of node *x*, node *x* is the jump point on the current search path.

This improved jump point screening strategy reduces the number of jump points in general.

### 3.3. Map Preprocessing

**Figure 7:**
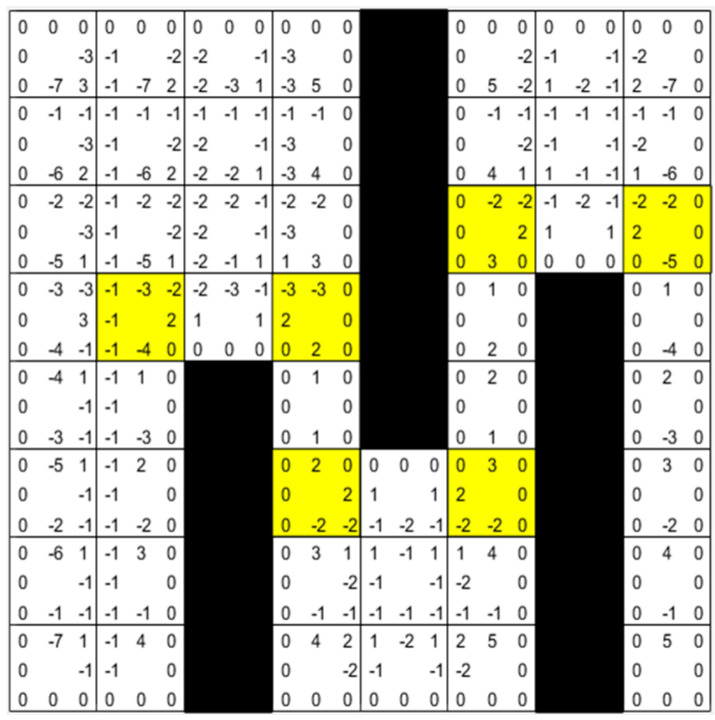
Preprocessed map. The yellow grid cells represent jump points.

The inflection points on the map are first filtered, and path planning is performed based on the relationship between the step lengths of each node’s eight directional extensions to reach the inflection points. Figure 7 shows an example of map preprocessing. The flowchart of map preprocessing is shown in Algorithm 1. Firstly, all the inflection points of the map are calculated, which are the yellow nodes. Next, the straight-line reachability of each node is evaluated, and the required step length to reach the jump point is recorded.

- A positive number *n* indicates a jump point that moves *n* steps in that direction to reach the inflection point or to search diagonally.
- A negative number *−n* indicates that moving *n* + 1 steps in that direction will hit an obstacle or boundary.
- A value of 0 means that the obstacle or boundary is encountered after moving 1 step in that direction.

At this point, the preprocessing process of the map is completed.
**Algorithm 1:** Map Preprocessing**Input:**grid map**Output:**map information data1**Function** map_preprocessing(map):2**for** free node n **in** map:3     **for** direction **do //**One of eight standard directions4        **if** reachable_jump_point **then**5            n.direction = step; //Return the distance to the jump point6        **else if** reachable_diagonal_special_point **then**7            n.direction = step; //Return the distance to the diagonal special point8        **else**9            n.direction = -step; //Return the step to obstacle or map boundary10        **end if**11     **end for**12      Processed_map←n;13**end for**14**return** Processed_map;

### 3.4. Improved Node Expansion

**Figure 8:**
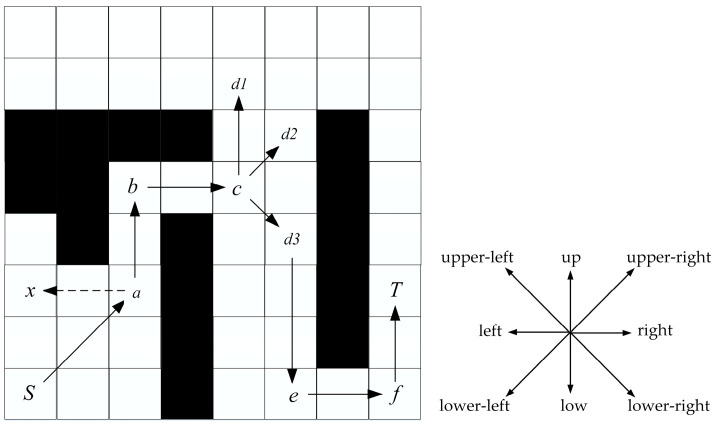
Node expansion.

Node expansion is the process of generating new nodes in a path search based on preprocessed map information. The node expansion of the JPS algorithm takes eight-neighborhood node expansion. However, the eight-neighborhood search is not used in the actual path search, which wastes the search memory as well as the search time. An eight-neighborhood search is proposed for the initial position, and a five-neighborhood search is recommended for the remaining intermediate nodes. Figure 8 shows the process of node expansion, where node *a* is obtained by expanding *S* in the upper-right direction, so node *a* will only expand in five directions: upper-left, upper-right, upper-right, right, and lower-right, and it will not expand to node *x*.

### 3.5. Bidirectional Path Search

**Figure 9:**
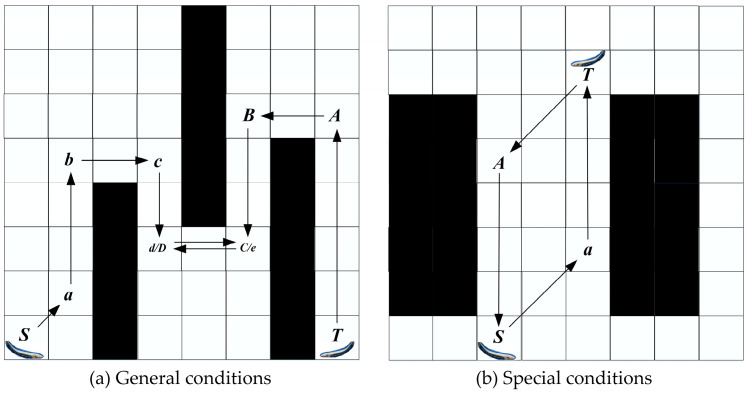
Path planning based on BJPS+ algorithm. *S* and *T* each represent an electric eel.

A bidirectional search performs a path search from both the start point and the target point. When the forward search reaches a node in the reverse search and the reverse search also reaches a node in the forward search at the same time, the path search will end and the forward and reverse paths will be combined to generate the final path. The bidirectional search process is shown in Algorithm 2. This strategy can effectively reduce the round-trip search for unnecessary nodes and improve the efficiency of the path [29].**Algorithm 2:** Bidirectional Path Search**Input:**start node S, target node T, processed map**Output:**a set of path nodes1**Function** bidirectional_path_search (S, T, map):2  open1←S; 3  open2←T;4  n1←S;5  n2←T;6  **while** n1 not in closed2 **or** n2 not in closed1 **do**7    n1←min_f in open1; //set current node;8    n2←min_f in open2; //set current node;9    open1.delete(n1);10    open2.delete(n2);11    closed1←n1;12    closed2←n2; 13    **if** n is reachable jump point form n1 **then //**forward path finding14      open1←{n}; **//**Expand node n based on preprocessed map15    **end if**16    **if** n is reachable jump point form n2 **then //**backward path finding17    open2←{n}; **//**Expand node n based on preprocessed map18    **end if**19  **end while**20  path←backtracking(closed1, closed2);21**return** path;

The process of bidirectional path planning is shown in Figure 9a, with *S* as the start point and *T* as the target point, and forward and reverse searching alternately. Firstly, the preprocessed map information is shown in Figure 7.

Step 1: Put *S* in Open List 1 for forward search and *T* in Open List 2 for reverse search.

Step 2: When searching forward, move 1 step up to the right because the parameter upper-right is +1, which can obtain node a and put a into Open List 1. When searching in the reverse direction, move up five steps because the parameter up is +5, which can obtain node *A* and put *A* into Open List 2.

Repeat the above operation until the sixth step, node *e* in the forward search is the point in the Closed List 2 of the reverse search, and node *D* is the point in the Closed List 1 of the forward search.

So far, the shortest path is found. We only need to fuse the positive and negative paths to obtain the final path, i.e., *S*-*a*-*b*-*c*-*d*(*D*)-*e*(*C*)-*B*-*A*-*T*. As in Figure 9b, in the special case of bidirectional search, if the path target point is searched first in one direction, the path search ends and the path is returned.

### 3.6. Rewiring Strategy

**Figure 10:**
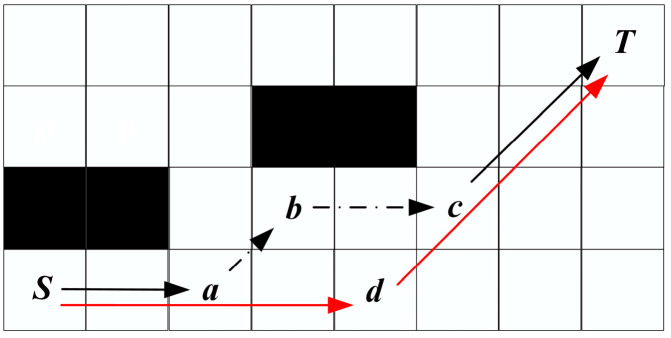
Parallelogram rewiring strategy.

Because the algorithm is set to only an eight-neighborhood search, the initially planned path contains redundant nodes. It not only increases the path length but also increases the number of turns between path nodes. Removing redundant nodes not only reduces the path length but also benefits the robot motion. The rewiring strategy is shown in Algorithm 3. In Figure 10, since the JPS takes a diagonal search-first strategy, the path-finding process will give priority to the inflection at node *a*, thus forming the inflection phenomenon of *S*-*a*-*b*-*c*-*T*.
**Algorithm 3:** Rewiring Strategy**Input:**path, map**Output:**optimized path1**Function** path_optimization (path, map):2  path←delect_same_direction_point(path); //Update path3  **for** i←1 **to** path.number-3 **do**
4    A.B.C.D.E←path(i): path(i + 4);5    **if** AB||CD **and**
$\vec{\mathrm{BC}}\cdot \vec{\mathrm{DE}}>0$ **then**6      N←chose_new_node(B, C, D); **//**BCDN is a parallelogram7      **if** safe(BN, DN) **then**8        **if** BC||DE **then //**case 19          path.delect(B, C, D);10        **else //**case 2: BC∩DE ≠ null11          path.delect(B, C);12        **end if**13      path.insert(N); //Insert node N after node A14      i←1;15      **end if**16    **end if**17  **end for //**Complete the Parallelogram Strategy18  path←path_discrete(path); //Update path19  path←path_prune(path); //Update path20**return** path

## 4. Simulation Studies and Discussion

In order to confirm the effectiveness of the algorithm in this paper, we simulate and compare the improved algorithm with four other representative algorithms in different sizes of grid maps, respectively. In addition, the improved algorithm is applied to the public dataset to confirm the effectiveness of the algorithm. Test machine is a 2.80 GHz Intel Core Duo processor with 8 GB of RAM running Windows 10.

### 4.1. Simulation of Different Algorithms

In this subsection, the A* [8], Bi-A* [29], JPS [22], JPS+ [23], and improved algorithms (without rewiring strategy) are simulated and compared to verify the effectiveness of the improved algorithms. The four algorithms are briefly described below. A* is the original algorithm. Bi-A*, JPS and JPS+ are variants of A*.

(1) A* is a heuristic search-based path-planning algorithm that selects the optimal path by taking into account heuristic functions and actual costs. It has a wide range of applications and is used in many areas to solve the shortest path problems.
(2) Bi-A* is an extension of the A* algorithm that simultaneously searches from both the start and end points to improve efficiency by simultaneously searching in both directions to find the shortest path.
(3) JPS is an improvement of the A* algorithm. It accelerates the search process by skipping unrelated intermediate nodes and considering only important nodes (called “jump points”). It leverages the continuous nature of the map to jump through path extensions, reducing unnecessary node extensions and improving the search efficiency.
(4) JPS+ is an improvement of the JPS algorithm. It includes preprocessing steps on the basis of the JPS algorithm to speed up the path search process by calculating and storing additional information beforehand. This preprocessing can be performed before the search, making the search process faster.

**Figure 11:**
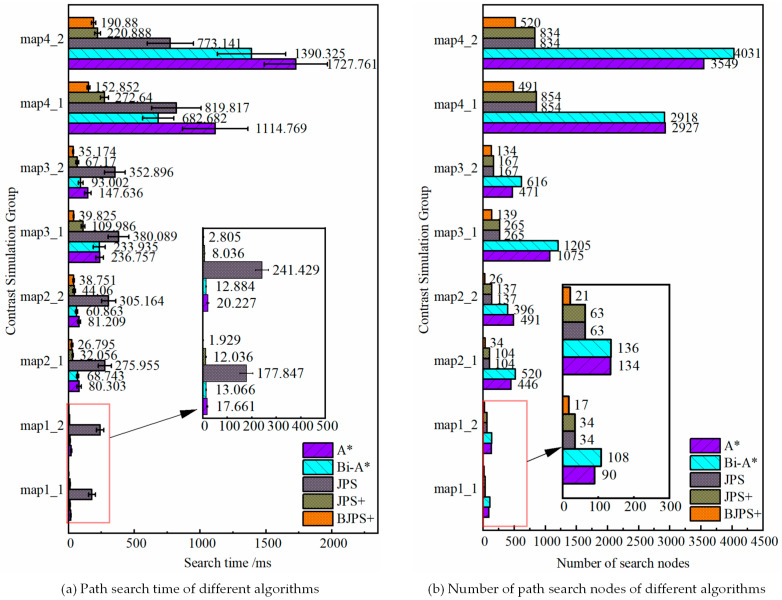
Comparison of simulation results of different algorithms.

**Figure 12:**
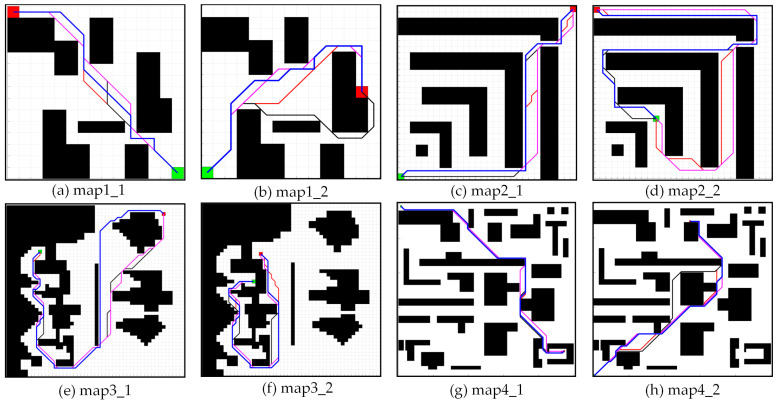
Simulation results. Paths planned by A* are shown in red. Paths planned by Bi-A* are shown in black. Paths planned by the JPS and the JPS+ are shown in purple. Paths planned by the BJPS+ are shown in blue.

Referring to the map sizes and obstacle distribution used in paper [30,31], the simulation uses map sizes of 15 × 15, 30 × 30, 50 × 50, and 100 × 100, and each size map takes a different obstacle distribution. The cost of moving in the straight direction is 1, and the cost of moving in the diagonal direction is $\sqrt{2}$. The simulation parameters are set in Table 1. Referencing article [1,31], we select three evaluation indicators. It include the path-planning time, the number of search nodes and the path length. Each group of experiments tests 20 times, and the path search time takes an average. Table 2 presents the simulation results data for different maps in our simulation group. We compare the time of path planning and the number of expanded nodes in the simulation group in Figure 11. Finally, the simulation results are shown in Figure 12.

Based on the simulation results and the taxonomy of the paper [32], the BJPS+ algorithm plans a definite path for a known map and a given start and target point. This behavior is deterministic and reproducible. According to the simulation results, it can be seen that the path search time of traditional A* is relatively short and the number of path search nodes is less in the small-size map. However, with the increase in map size, the search time and the number of path search nodes of A* will increase sharply. Bi-A* shows better search time performance in large-scale maps and has advantages over traditional A*. However, the process of generating jump points by JPS requires a large amount of memory space and computational resources, and its performance is slightly lower than that of A* in small size maps, and it may have the problem of over-searching. The BJPS+ proposed in this paper shows better performance than the other four algorithms in terms of the search time and number of search nodes, and the algorithm is not affected by the map size or obstacle complexity. In terms of path length, the BJPS+ plans a path that is approximately 4.37% longer than variants of A*. The increased path cost is due to the improved jump point screening strategy used in the map preprocessing process. It can avoid diagonals which graze corners of obstacles. This improvement is good for driving safety. Meanwhile, BPS+ has about 43.31% less path planning time than the best of the other four algorithms. And BPS+ has about 51.70% fewer path search nodes than the best of the other four algorithms. Therefore, a small increase in path length is acceptable.

To further prove the effectiveness of the BJPS+ algorithm, maps with different obstacle densities are taken for experiments. All map sizes are 50 × 50. Each obstacle density map generates 20 maps by randomization. Two sets, each including a start point and target point, were used for each map. Each set of experiments was repeated 20 times. The experimental results of the same obstacle density maps are summarized and processed. The path planning length, path search time and map preprocessing time are averaged. And the number of expansion nodes is rounded to the nearest whole number after averaging. The experimental results are shown in Table 3.

The experimental results show that as the density of obstacles increases, BJPS+ has a significant disadvantage in path planning in maps with a large distribution of small obstacles (consisting of 1–3 grid cells). When the density of obstacles increases, the BJPS+ algorithm will plan about 10% more path lengths than the other four algorithms, but the time may decrease very little (5–10%). Even in a small number of map experiments, the path search time of the BJPS+ may be longer than that of A*. This is due to the presence of many grid-cell sized individual obstacles in the map. But in real maps, an obstacle occupies dozens or even more grid cells. And the map is inflated with obstacles before the actual path planning. This removes a large number of tiny obstacles. Therefore, the advantage of the BJPS+ is magnified when used in practice. In addition, the improved map preprocessing algorithm is about 95% faster than the original algorithm. In general, the BJPS+ algorithm performs quite well.

### 4.2. Simulation of Public Data Sets

**Figure 13:**
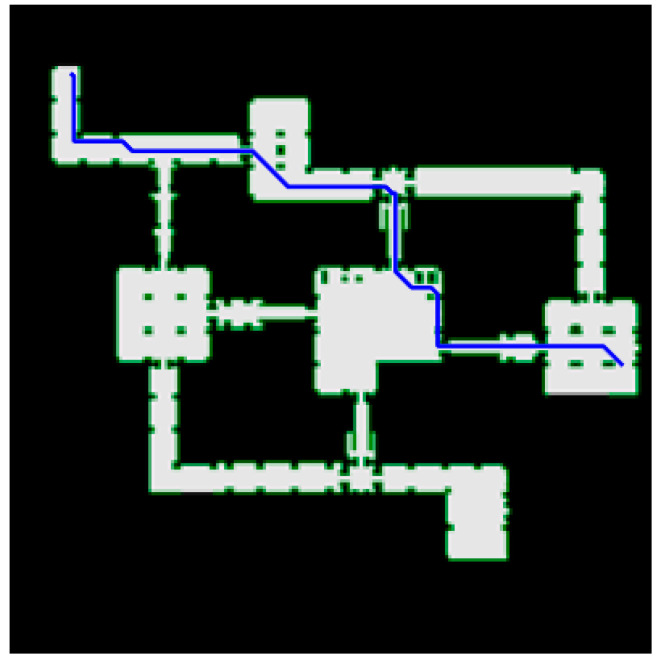
The improved algorithm is applied to dr *slavers map*. The map origin is in the lower left corner, the starting point is (107, 966), and the target point is (1020, 483). The path search time is 483.857 ms, the path length is 1162.118, and the number of search nodes is 1135.

The improved algorithm is applied to the game <*Dragon Age II*>. The dataset takes dr_slavers map, which Sturtevant [33] provides free of charge. The map size is 1073 × 1073, and the simulation results are shown in Figure 13.

**Figure 14:**
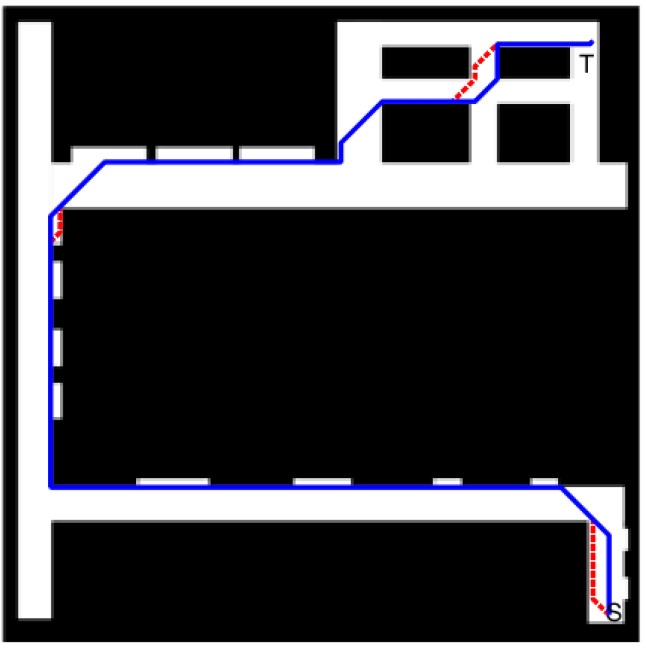
The improved algorithm is applied to dr *daheng3 map*. The red path is obtained without a rewiring strategy, and the blue path is obtained using a rewiring strategy. The map origin is in the lower left corner, the starting point is (1900,100), and the target point is (1850,1900). The path search time is 170.616 ms, the path length is 4746.830, and the number of search nodes is 371.

To evaluate the performance of the improved algorithm for path planning in structured occasions, we used an experimental floor *daheng3 map* with a map size of 2000 × 2000. The simulation results are shown in Figure 14, and the results show that the improved algorithm has a huge advantage in the two evaluation metrics of the search time and the number of search nodes in structured occasion search. In addition, the optimized path using the parallelogram rewiring strategy ensures a maximum straight-line travel distance. It ensures a faster travel speed for the robot. The reduction in inflection points ensures that the robot travels safely.

## 5. Conclusions

An improved bidirectional JPS algorithm is proposed in this study based on the electricity-guided navigation behavior of electric eels and map preprocessing. By drawing on the electric eel’s ability to use electric fields in water to quickly find its companions, this study has successfully improved the robot’s ability to locate and navigate in specific environments. The aim of this study is to reduce the path search time and storage memory by introducing an improved heuristic function, a new jump point screening strategy, a anode expansion strategy, and a bidirectional search strategy. Additionally, a rewiring algorithm is developed to reduce the number of inflection points. The combination of these five improvements has resulted in significant performance enhancements for the path search algorithm. Furthermore, experimental results on a public dataset demonstrate the effectiveness of the improved algorithm. Overall, the findings indicate that the proposed algorithm can be successfully applied in complex situations, particularly in practical structured scenarios.

## Figures and Tables

**Table 1:** Parameter settings of algorithm simulation.

Map Sizes	Map Types	Simulation Groups	Start	Target
15 × 15	simple	map1_1	(15, 1)	(1, 15)
		map1_2	(1, 1)	(14, 8)
30 × 30	promenade	map2_1	(1, 1)	(30, 30)
		map2_2	(11, 11)	(1, 30)
50 × 50	complex	map3_1	(10, 37)	(45, 48)
		map3_2	(15, 28)	(17, 36)
100 × 100	complex	map4_1	(2, 99)	(95, 15)
		map4_2	(1, 1)	(55, 90)

**Table 2:** Simulation results of different algorithms.

Simulation Groups	Algorithm	Evaluation	map1_1	map1_2	map2_1	map2_2	map3_1	map3_2	map4_1	map4_2
Search time/ms	A*	Mean	17.661	20.227	80.303	81.209	236.757	147.636	1114.769	1727.761
		Error margin	1.432	1.757	7.218	4.465	13.039	10.794	108.897	105.054
	Bi-A*	Mean	13.066	12.884	68.743	60.863	233.935	93.002	682.682	1390.325
		Error margin	0.927	0.717	3.474	3.571	20.420	7.622	51.807	113.319
	JPS	Mean	177.847	241.429	275.955	305.164	380.089	352.896	819.817	773.141
		Error margin	11.449	11.869	21.608	23.582	34.621	34.247	81.846	76.790
	JPS+	Mean	12.036	8.036	32.056	44.06	109.986	67.17	272.64	220.888
		Error margin	0.891	0.579	1.925	3.963	6.035	5.227	13.230	9.794
	BJPS+	Mean	1.929	2.805	26.795	38.751	39.825	35.174	152.852	190.88
		Error margin	0.345	0.334	2.642	2.075	1.434	2.283	4.928	6.803
Number of Search nodes	A*	/	90	134	446	491	1075	471	2927	3549
	Bi-A*	/	108	136	520	396	1205	616	2918	4031
	JPS	/	34	63	104	137	265	167	854	834
	JPS+	/	34	63	104	137	265	167	854	834
	BJPS+	/	17	21	34	26	136	134	491	520
Path length	A*,Bi-A*	/	21.556	22.728	53.314	74.556	102.569	75.113	155.711	141.51
	JPS, JPS+	/	21.556	22.728	53.314	75.971	102.569	75.113	155.711	141.51
	BJPS+	/	23.314	23.9	55.071	78.9	106.856	77.456	158.642	146.197

The search time is averaged over 20 experiments. The comparison of path search times for each experiment can be got from Supplementary Materials. Error margin represents range with a confidence level of 95%. Those can also be got from Supplementary Materials.

**Table 3:** Summary of map experiments with different obstacle densities.

Obstacles (%)	Algorithm	Search Time/ms	* (%)	Number of Search Nodes	* (%)	Path Length	* (%)	Map Preprocessing Time/s	* (%)
5	A*	45.966 (0.572)	−71.78	360	−66.94	70.117	7.84	/	/
5	Bi-A*	44.927 (2.371)	−71.13	356	−66.57	70.117	7.84	/	/
5	JPS	55.940 (3.002)	−76.81	153	−22.22	70.117	7.84	/	/
5	JPS+	15.201 (1.666)	−14.67	153	−22.22	70.117	7.84	9.798	−97.07
5	BJPS+	12.971 (1.806)	/	119	/	75.614	/	0.287	/
10	A*	49.383 (0.859)	−39.40	488	−50.00	70.864	11.25	/	/
10	Bi-A*	46.826 (3.783)	−36.09	485	−49.69	70.864	11.25	/	/
10	JPS	86.280 (2.395)	−65.31	254	−3.94	70.864	11.25	/	/
10	JPS+	37.232 (1.519)	−19.62	254	−3.94	70.864	11.25	8.264	−97.12
10	BJPS+	29.927 (2.085)	/	244	/	78.835	/	0.238	/
20	A*	77.826 (1.615)	−7.04	684	−39.33	73.008	12.95	/	/
20	Bi-A*	78.832 (3.919)	−8.23	747	−44.44	73.008	12.95	/	/
20	JPS	196.747 (3.889)	−63.23	446	−6.95	73.008	12.95	/	/
20	JPS+	87.594 (2.548)	−17.41	446	−6.95	73.008	12.95	5.354	−96.25
20	BJPS+	72.348 (1.755)	/	415	/	82.464	/	0.201	/
30	A*	97.593 (2.118)	−11.96	779	−36.33	75.914	11.29	/	/
30	Bi-A*	95.360 (3.757)	−9.90	741	−33.06	75.914	11.29	/	/
30	JPS	229.829 (4.857)	−62.62	517	−4.06	75.914	11.29	/	/
30	JPS+	89.223 (2.145)	−3.71	517	−4.06	75.914	11.29	3.758	−95.32
30	BJPS+	85.917 (1.432)	/	496	/	84.481	/	0.176	/
40	A*	64.435 (1.535)	−4.31	800	−50.00	82.560	12.61	/	/
40	Bi-A*	107.793 (3.154)	−42.80	680	−41.18	82.560	12.61	/	/
40	JPS	219.032 (4.471)	−71.85	478	−16.32	82.560	12.61	/	/
40	JPS+	78.156 (1.333)	−21.11	478	−16.32	82.560	12.61	2.691	−94.35
40	BJPS+	61.656 (1.098)	/	400	/	92.968	/	0.152	/

It indicates the percentage increase in BJPS+ metrics over the corresponding algorithms. In the search time column, such as A (B), A represents the mean, and B represents the mean of error margins for 20 sets of experiments (confidence level of 95%). The error margins of the path search time for each set of experiments can be got from Supplementary Materials (not include a certain confidence value).

## Data Availability

All relevant data are within the paper.

## Supplementary Materials

The following supporting information can be downloaded at: https://www.mdpi.com/article/10.3390/biomimetics8050387/s1, Figure S1: comparison of path search times for each experiment in Table 2; Table S1: error margins of 95% of the path search time for each set of experiments in Table 2; Table S2: SD1.xlsx, SD2.xlsx, SD3.xlsx, SD4.xlsx, SD5.xlsx contains the experimental results (standard deviation) of A *, Bi-A *, JPS, JPS +, BJPS + algorithms respectively. Sheet1, Sheet2, Sheet3, Sheet4, Sheet5 corresponding barrier coverage 5%, 10%, 20%, 30%, 40%. The data was not subjected to the calculation of error margins of confidence for specific values.
