# Supplementary figures and images for: Bidirectional Jump Point Search Path-Planning Algorithm Based on Electricity-Guided Navigation Behavior of Electric Eels and Map Preprocessing
Source: Biomimetics (Basel). 2023 Aug 25;8(5):387. doi: 10.3390/biomimetics8050387 (PMC10526936; doi:10.3390/biomimetics8050387)

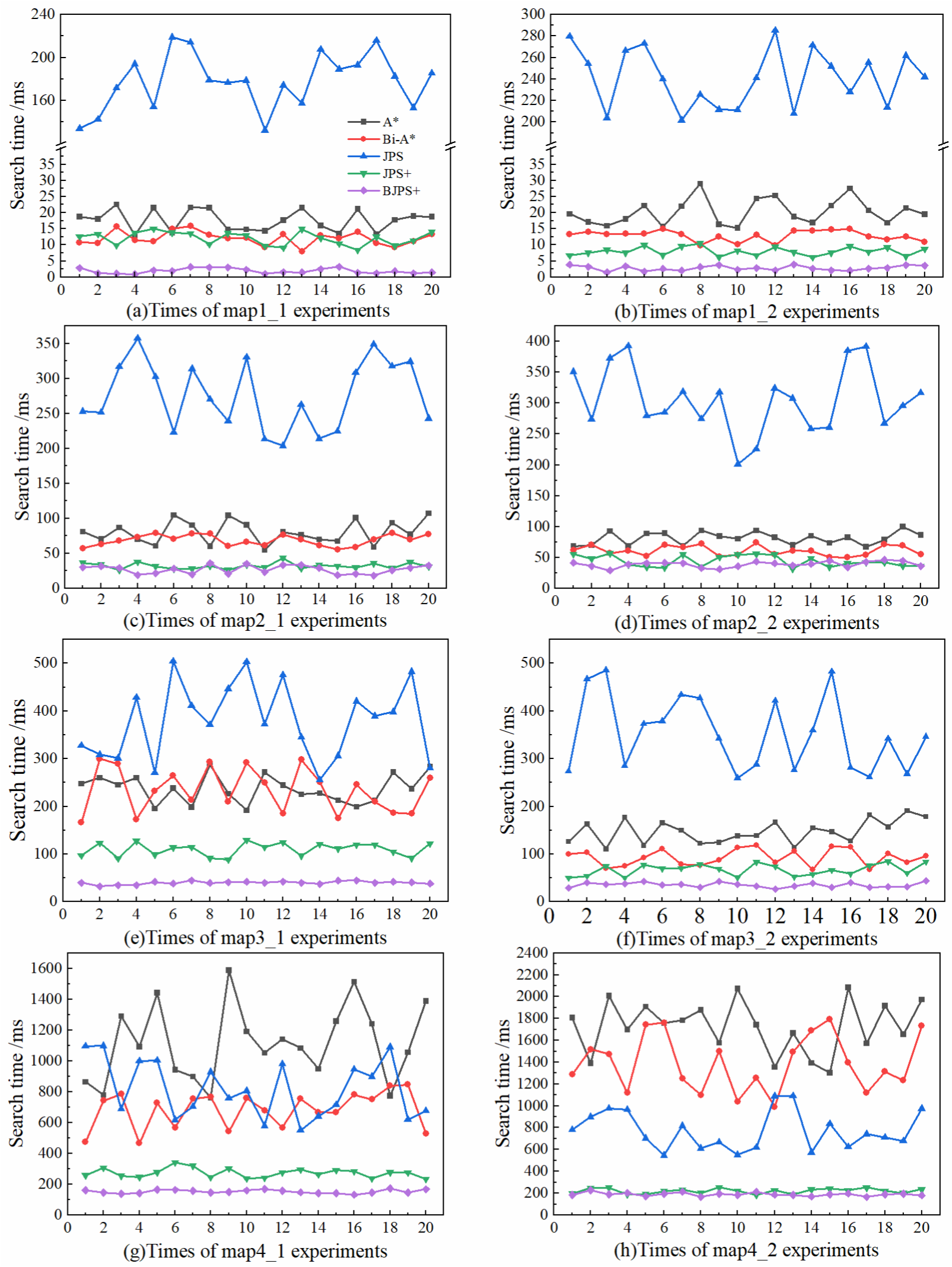

Supplement: Supplementary file 1 [file biomimetics-08-00387-s001.zip › figure S1.tif]
